# Field data on plant growth and insect damage on the noxious weed *Solanum eleaegnifolium* in an unexplored native range

**DOI:** 10.1016/j.dib.2018.07.022

**Published:** 2018-07-11

**Authors:** Rupesh R. Kariyat, Jesus Chavana

**Affiliations:** Department of Biology, University of Texas Rio Grande Valley, Edinburg, TX 78539, United States

## Abstract

In this data article, we provide a novel data set on plant growth, insect damage levels, and herbivore community of the noxious and invasive weed *Solanum eleaegnifolium* (Solanaceae). The data is collected from disturbed and un-disturbed urban populations of the species from one of its unexplored native range in Southern United States (South Texas). The data include plant height measurements, insect damage levels, GPS coordinates of the populations, and their disturbance status. Additional data includes the number of chewing herbivore (specialist herbivore Texas potato beetle (*Leptinotarsa texana*; Chrysomelidae), their eggs, and any lepidopteran caterpillars found on the plants.

**Specifications Table**

**Table:** 

Subject area	Biology
More specific subject area	Invasion ecology, Entomology
Type of data	Table, figures, pictures, and maps
How data was acquired	Survey and observation
Data format	Raw, filtered and analyzed
Experimental factors	Sampled populations were designated into disturbed and non disturbed
Experimental features	Native populations in urban and semi-urban area
Data source location	McAllen, Edinburg, Texas, USA (GPS coordinates in the excel file)
Data accessibility	Data available as attached with the manuscript

**Value of the data**

• First data set on plant traits from the South Texas native populations of *Solanum eleaegnifolium*, a noxious weed that is invasive worldwide.
• Data set provides population level data on disturbance, plant growth, and defense for meta analyses.
• Dataset also provides count data on major insect herbivores of two different feeding guilds that would make excellent addition to worldwide data on functional and defense traits of *Solanum eleaegnifolium*, and similar weeds.

## 1 Data

We provide and extensive survey and observation based data on growth and defense traits of *Solanum eleaegnifolium*, from disturbed and undisturbed populations in its native range in South Texas. Plant height, population type, and insect data are presented in Supplementary Table 1, while details of the populations are presented in Supplementary Table 2.

## 2 Experimental design, materials and methods

**Fig. 1:**
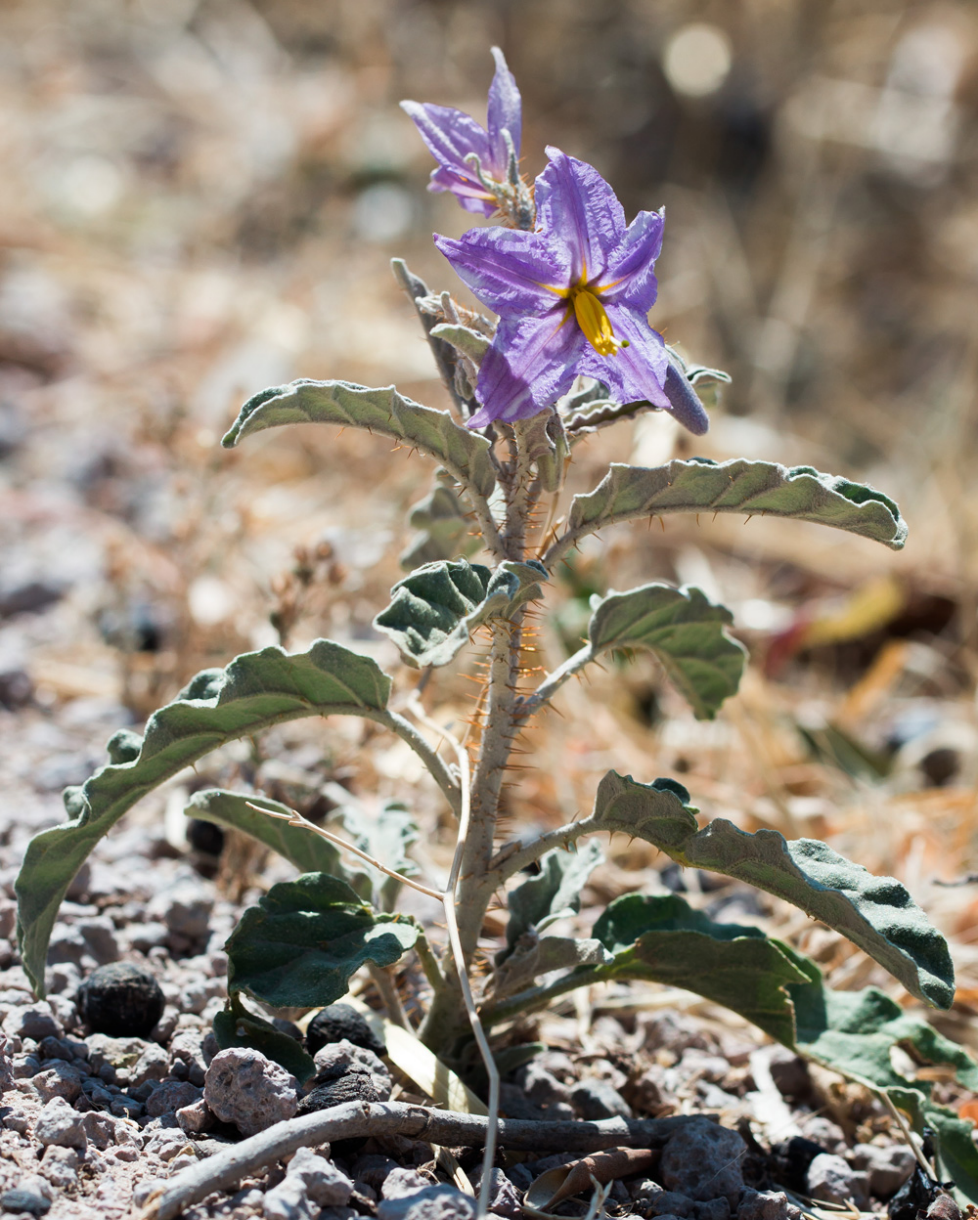
A *Solanum eleaegnifolium* plant in bloom.

The experiment was designed to collect data from multiple populations of silver leaf nightshade (*Solanum eleaegnifolium*) from one of its major native ranges- South Texas. *S. eleaegnifolium* is a perennial herb native to southwest, west-central USA and Northern Mexico ([1], [2]; Fig. 1). *S. eleaegnifolium* colonize disturbed areas, outcompete co-inhabiting plants for resources, and have both structural (spines, trichomes) and chemical defenses (secondary metabolites that are toxic to livestock), making it hard to eradicate [2], [3], [4]. The species has colonized all over the world and is a grave concern in many countries. Although data on growth and other functional traits of this species has been collected from other native and invasive ranges, we currently lack data from one of its major native range in South Texas [2].

We sampled urban and semi-urban populations, and data was collected on plant height, scale of insect damage, types of insects feeding, and their numbers (Supplementary Table 1). Data on GPS coordinates was also collected for each of the populations (Supplementary Table 2). A minimum of 10 genets per population was sampled. Care was taken to ensure that the genets were at least few meters apart to not sample clones, as the species can propagate through rhizomes. Height was measured to the 6 closest centimeters. Insect damage was measured on a scale from 0 to 5, with zero- plants having no damage, 1 having minor damage on one or two leaves, 2- damage on 25% of the leaves, 3- on 50%, 4-on 75%, and 5- the plant completely damaged and with no leaves without damage (for details see [5]). Then we followed up on examining the kind of insect damage by counting the number of herbivores feeding on them. Data on number of caterpillars if any, number of beetles, and their eggs, was also collected. The populations were also GPS mapped and given a location name to be able to go back later in the season to collect additional data (Supplementary Table 2). We plan to follow up the current data set by collecting fruits and seeds at the end of the growing season.

**Fig. 2:**
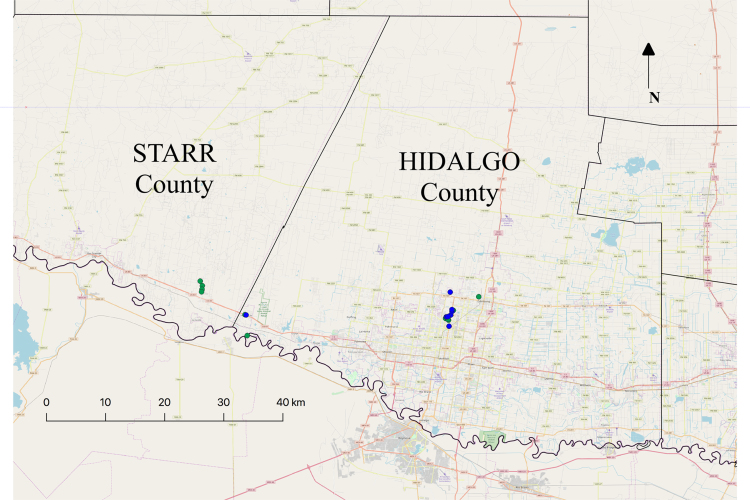
QGIS based map of the counties in south Texas used for data collection.

The GPS coordinates that represent disturbed and undisturbed populations on the map were plotted using Microsoft excel and QGIS 3.0 (Open Source Geographic Information System). First the raw data from the GPS device (My GPS coordinates android application on Samsung Galaxy S8 smart phone) was input into excel either in DMS (degree minutes seconds) or in decimal format, and then saved in csv file format. To interpret the data through QGIS, first a map of the desired area (Starr and Hidalgo counties) were to be downloaded and then uploaded to QGIS. This was carried out using map resources from txdot.gov that show the county boundaries layer. QGIS has a plugin for uploading such maps (e.g., Google maps, Bing and MapQuest) which were then added as a map layer that show a street view. After adding the map layers, the.csv file with the raw data was superimposed along with any additional information (e.g., labels, shapes and colors) as required. In the current study, we used different colors (same shape) to represent disturbed and undisturbed populations. Afterwards, the maps were exported into the desired file type to be saved for representation in the manuscript (Fig. 2).

## Footnotes

Appendix A Supplementary data associated with this article can be found in the online version at https://doi.org/10.1016/j.dib.2018.07.022.
