# Supplementary material for: Field data on plant growth and insect damage on the noxious weed Solanum eleaegnifolium in an unexplored native range
Source: Data Brief. 2018 Jul 11;19:2348–51. doi: 10.1016/j.dib.2018.07.022 (PMC6141521; doi:10.1016/j.dib.2018.07.022)
Supplement: Supplementary file 1 — Supplementary material [file mmc1.docx]

Conflict of interest:

The authors declare that there is no conflict of interest.
